# Supplementary material for: A strategy to quantify myofibroblast activation on a continuous spectrum
Source: Sci Rep. 2022 Jul 18;12:12239. doi: 10.1038/s41598-022-16158-7 (PMC9293987; doi:10.1038/s41598-022-16158-7)
Supplement: Supplementary file 1 — Supplementary Information 1. [file 41598_2022_16158_MOESM1_ESM.pdf]

Supplementary Information

A Strategy to Quantify Myofibroblast Activation on a Continuous Spectrum

Alexander Hillsley, Matthew S. Santoso, Sean M. Engels, Kathleen N. Halwachs, Lydia M. Contreras, Adrianne M. Rosales*

	Activated		Non-Activated	
**Feature**	**avg**	**error**	**avg**	**error**
Cell Area	22631.89	13733.01	4002.48	3166.87
Cell Perimeter	1397.04	674.56	410.99	241.26
Cell Major Axis Length	242.41	75.55	113.70	56.03
Cell Minor Axis Length	152.39	59.06	53.46	25.08
Cell Circularity	0.17	0.10	0.27	0.10
Cell Eccentricity	0.71	0.17	0.82	0.14
Cell Extent	0.46	0.13	0.46	0.13
Nucleus Area	485.79	319.75	189.92	96.03
Nucleus Perimeter	86.94	39.45	53.32	15.27
Nucleus Major Axis Length	29.14	12.15	19.27	5.12
Nucleus Minor Axis Length	19.21	8.48	12.09	3.42
Nucleus Circularity	0.69	0.24	0.80	0.13
Nucleus Eccentricity	0.68	0.21	0.75	0.12
Nuc Cyt Ratio	0.02	0.01	0.04	0.01
Minkowski-Bouligand dimension	1.16	0.08	1.09	0.09
Mean Pearson's r whole	0.24	0.29	-0.14	0.25
Mean Pearson's r 64x64	0.47	0.20	0.11	0.20
Mean Pearson's r 32x32	0.49	0.18	0.19	0.15
Mean Pearson's r 16x16	0.48	0.18	0.20	0.13
Mean Pearson's r 8x8	0.44	0.17	0.18	0.11
Mean Pearson's r 4x4	0.37	0.16	0.13	0.09

Table S1. Average value and standard deviation of all measured cell features. Units are in μm for perimeters and axis lengths, μm^2 for areas, and unitless for all other features.

Measured Cell Feature Descriptions

Area, perimeter, major/minor axis, eccentricity, and extent were all measured using the scipy.measure.regionprops python function. More information can be found in their documentation.

1. Cell/Nucleus Area: The number of pixels in the mask, multiplied by the size of each pixel

2. Cell/Nucleus Perimeter: Approximated, by drawing a line through the center of each border pixel in the mask

3. Cell/Nucleus Major/Minor Axis Length: the major or minor axis of an ellipse fit to the mask

4. Cell/Nucleus Circularity: The ratio of mask area, to the area of a circle with the same perimeter as the mask. This gives a measure of how close to a perfect circle the cell shape is. A perfect circle has a circularity of 1 while a line has a circularity of 0

5. Cell/Nucleus Eccentricity: The eccentricity of an ellipse fit to the mask. The eccentricity is the ratio of the focal distance (distance between focal points) over the major axis length.

6. Cell/Nucleus Extent: The ratio of the area of a cell divided by the size of the smallest possible bounding box, or rectangle that can fully encompass the cell. Cell extent is a measure of how spread out a cell is

7. Nuc / Cyt ratio: The ratio of the area of the nucleus to the area of the cell.

8. Minkowski-Bouligand Dimension: A method of estimating the fractal dimension of the cell mask. This provides a measure of how rough the cell boundary is.

9. Mean Pearson's R: The Pearson's correlation coefficient between the a-SMA (green) and F-Actin (red) channels of the image. Pearson's R must be calculated over multiple pixels, therefore the image was first broken down into tiles of various sizes (4, 8, 16, 32, and 64 pixels). Pearson's R was then calculated for each tile, and all the tiles in an image averaged. A value of 0 corresponds to random / no correlation, while a value of 1 corresponds to perfect correlation.

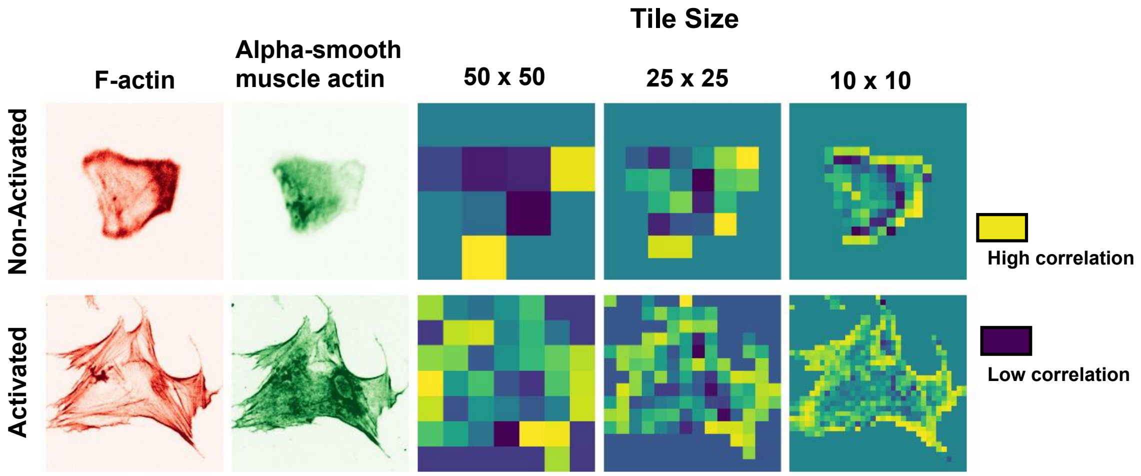

Figure S1. Colocalization of F-actin and alpha-SMA was quantified using the Pearson's correlation coefficient (R_P). In Python, the image was first broken into a series of small tiles (Tile sizes of 50 x 50, 25 x 25, and 10 x 10 pixels shown). R_P was then calculated for each tile (yellow – high colocalization, purple – low colocalization). All cell containing tiles were then averaged to calculate an average colocalization for each cell. On average, activated cells had a significantly higher degree of colocalization across all tile sizes.

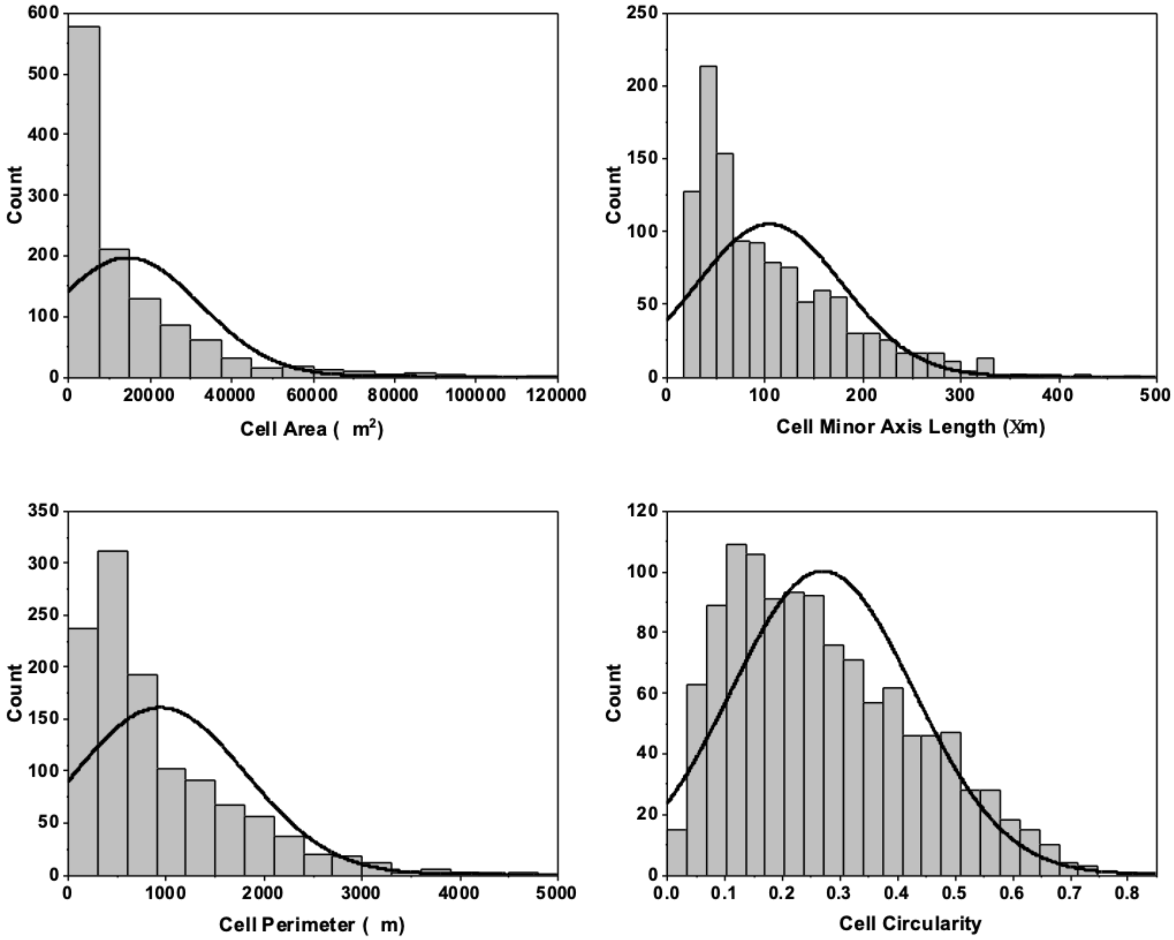

Figure S2. Histograms of four measured cell features for all 1170 cells. Without manually separating activated myofibroblasts from non-activated fibroblasts, the cells appear to be a part of a single distribution.

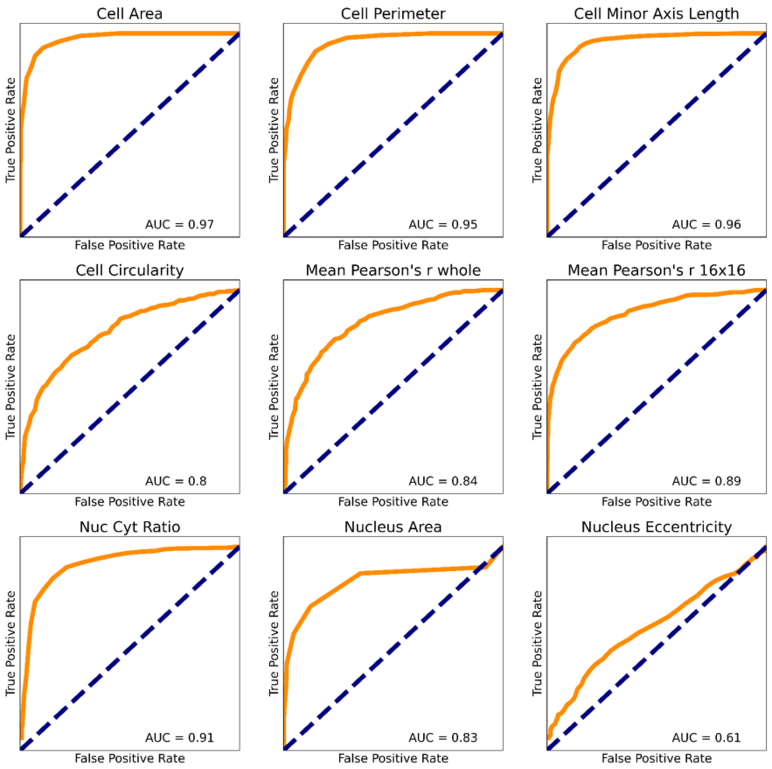

**Property**	**AUC**
Cell Area	0.97
Cell Minor	0.96
Cell Perimeter	0.95
Cell Major	0.92
Nuclear/Cytosolic ratio	0.91
Pearson's R 64x64	0.90
Pearson's R 8x8	0.89
Pearson's R 32x32	0.89
Pearson's R 4x4	0.89
Pearson's R 16x16	0.89
Pearson's R whole	0.84
Nuclear Area	0.83
Nuclear Major	0.82
Nuclear Minor	0.82
Nuclear Perimeter	0.81
Cell Circularity	0.80
Minkowski–Bouligand dimension	0.72
Cell Eccentricity	0.69
Nuclear Circularity	0.66
Nuclear Eccentricity	0.61
Cell Extent	0.51

Figure S3. ROC curves for nine measured cell features. AUC values were calculated from ROC curves for all 21 features and are shown in the table.

Decision Tree

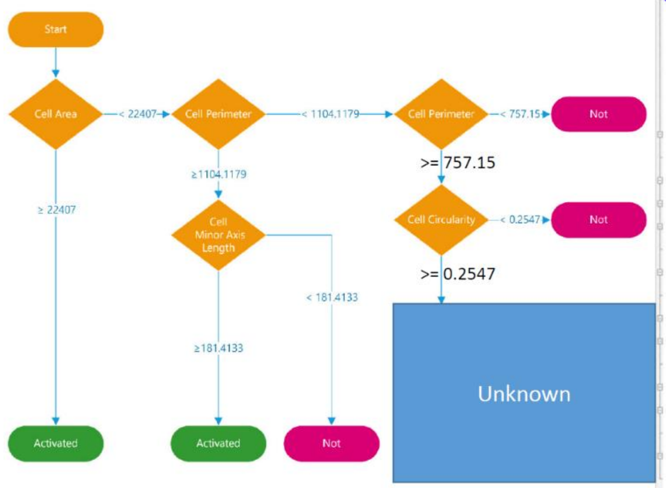


```python
def run_dTree_3(CellArea, CellPeri, CellMinorLength, CellCirc):
    '''Here lies tree combining both cell area and no cell area trees
    has low error, 11% for activation, 5% for not
    gets about 7% error rate
    see: fPredTree3.3.png for real tree
    original: fPredTree3 for other tree options'''
    if CellArea >= 22407:
        return "Activated"
    else:
        if CellPeri >= 1104.117928:
            if CellMinorLength >= 181.413255:
                return "Activated"
            else:
                return "Not"
        else:
            if CellPeri < 757.15:
                return "Not"
            else:
                if CellCirc < 0.254725636:
                    return "Not"
                else:
                    return "Unknown"
```

Figure S4. Illustration of the decision tree model and the “if” statements used in its code.

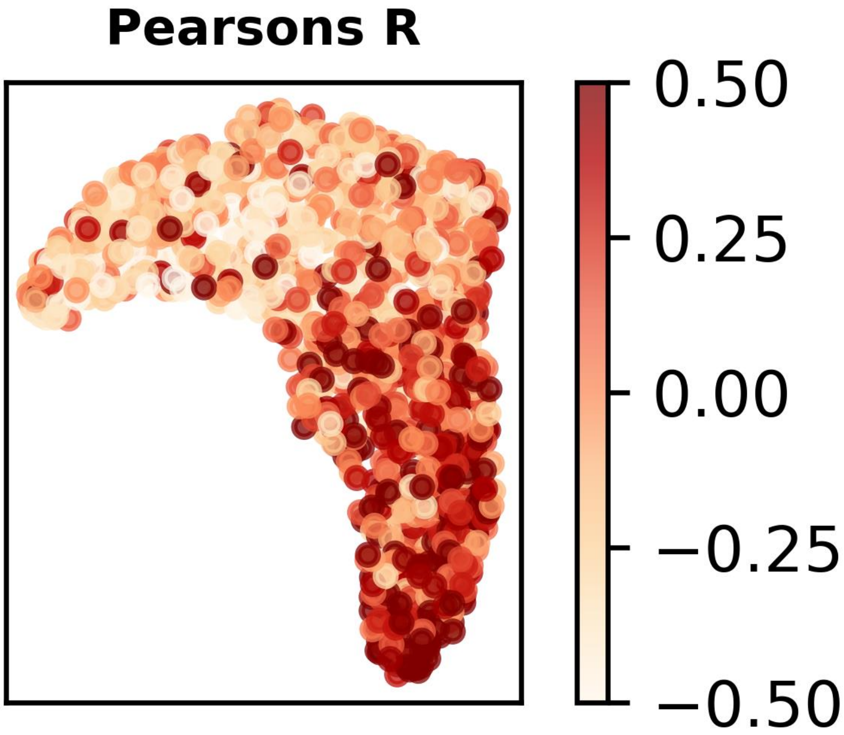

Figure S5. To further compare our classification models to traditional measures of activation (a-SMA stress fiber formation), heatmaps were generated with individual cells colored by their Pearson's R coefficient.

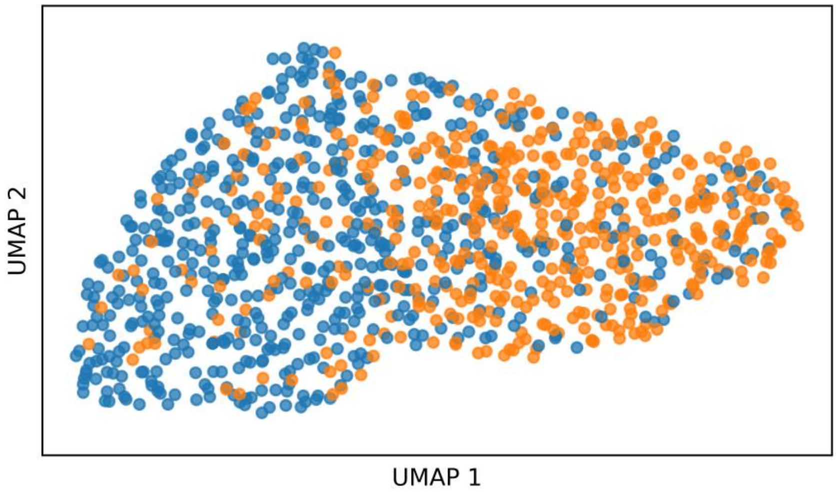

Figure S6. A traditional measure of myofibroblast activation is the association of a-SMA into stress fibers. In order to demonstrate that our manually engineered model agrees with this traditional classification method, another UMAP and PCA plot were constructed, containing the Pearson's R coefficient for the whole cell, in addition to the 4 properties listed in Figure 2A. This UMAP reduction shows a similar spectrum of activation, this time along the UMAP 1 axis. Specific axes have no meaning in UMAP reductions, so this trend matches that in figure 2C.

A)

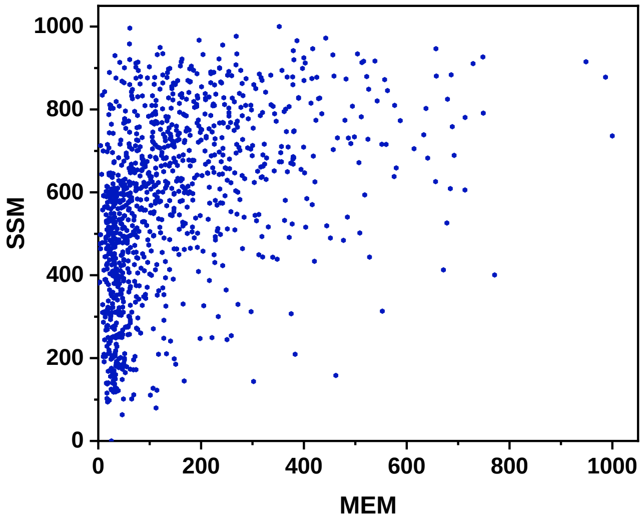

B)

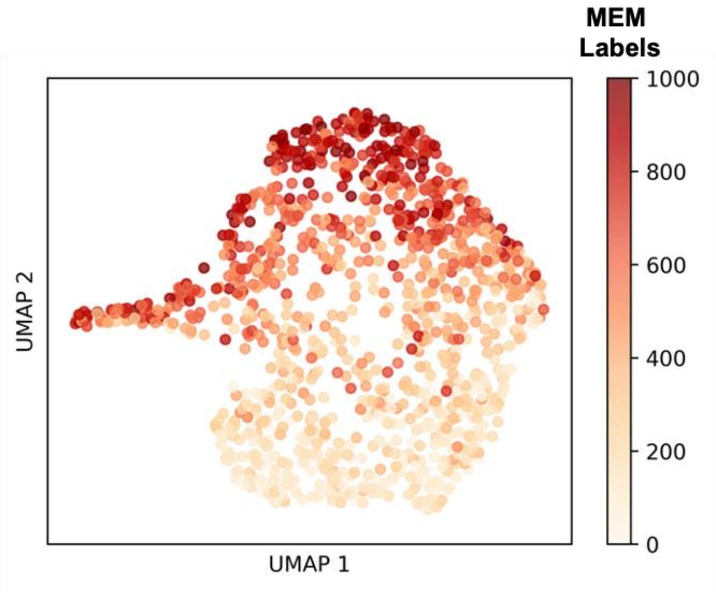

Figure S7. Comparison of the SSM and MEM label systems. A) SSM provides a relatively even distribution of labels between 0 and 1,000, while the MEM labels are clustered at the lower end of the spectrum, with very few cells labeled > 800. B) MEM labels imposed on the SSM UMAP.

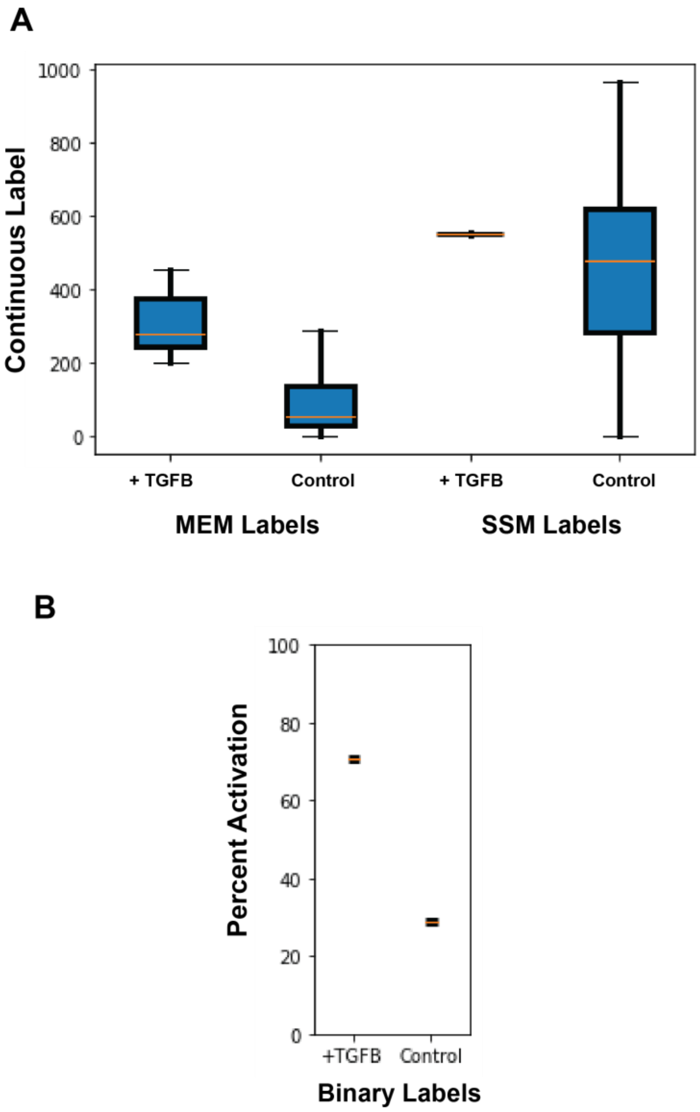

Figure S8. As a proof of concept, cardiac fibroblasts were cultured in TGFB+ media (complete DMEM, 10% FBS, 1% PS, 10 ng/mL TGFB), fixed, stained, and pushed through the same analysis pipelines (Figures 2A and 3A), to assign them labels in the same continuous systems reported in Figures 2 and 3. More information on this analysis can be found on the Github page. In total 24 cells, grown in TGFB+ media, were analyzed. The control group for this study, 500 cells, imaged across 3 separate cultures, from the original dataset (Figure 1A). Only the first 3 separate cultures were used, because they captured the true ratio of activated to non-activated cells. A) Both the MEM and the SSM capture an increase in average percent activation with the addition of TGFB, although the SSM model shows only a slight increase. B) The binary or traditional labeling system shows a significant increase in activation

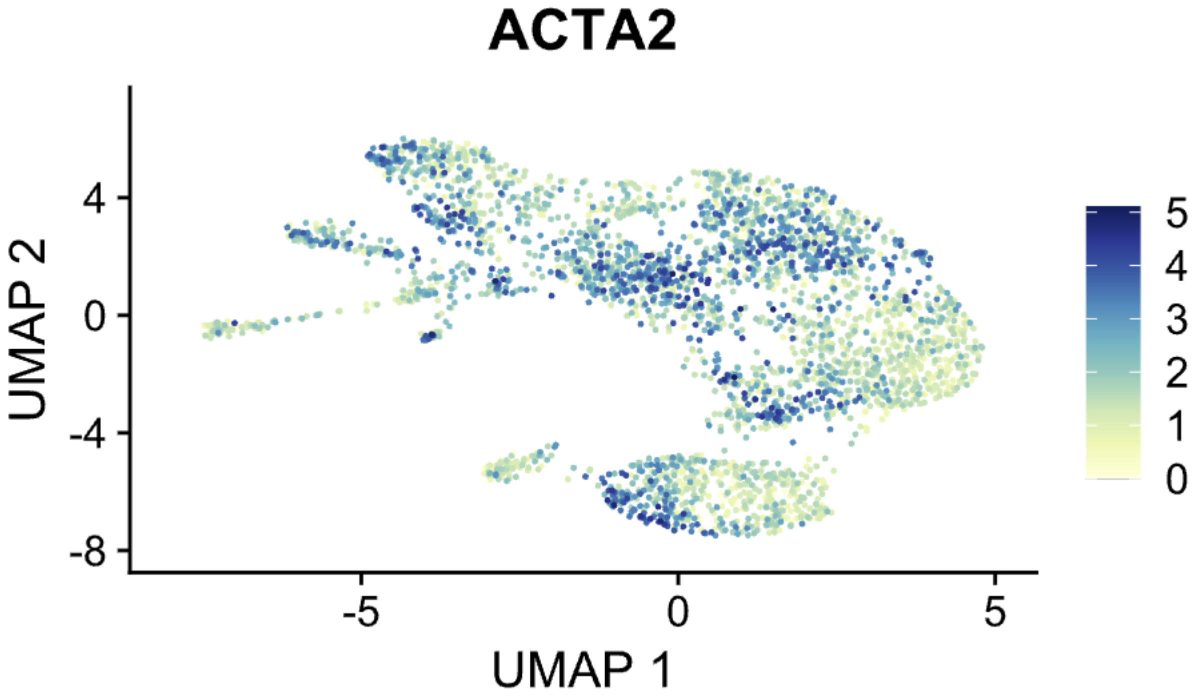

Figure S9. UMAP reduction of scRNA-seq analysis of 4,062 cells. Each individual cell is colored by its normalized expression levels of ACTA_2, the gene responsible for a-SMA production. Darker colors represent higher expression levels. Although less differentially expressed, this heatmap shows similar trends with other myofibroblast associated genes, increasing in the negative UMAP 1 direction.

PC 1		PC2	
**Up-Regulated**	**Down-Regulated**	**Up-Regulated**	**Down-Regulated**
MT2A	MALAT1	PTX3	S100A16
KRT18	GAS5	ANKRD1	S100A6
TIMP1	S100A16	CCN2	AKR1C1
UACA	FGF2	ADAMTS1	AKR1C3
POSTN	RALA	FGF2	S100A4
CRYAB	MFAP5	MALAT1	MFAP5
IGFBP5	DDIT3	PPME1	TMEM158
PLAT	THBS1	TNFRSF11B	GAPDH
TFPI2	VIPR1	THBS1	S100A10
PRSS23	NIBAN1	INHBA	NRP2
LGALS1	ZFAS1	TPM1	TIMP1
MYL9	SH3BGR	KRT18	FTL
SPARC	NRP2	SERPINE1	AKR1C2
GAPDH	PTX3	PCDH10	VIM
HSPB6	TGFB2	CALD1	FTH1
SH3BGRL3	HSPA5	COL8A1	RARRES2
TPM2	AKR1C1	MMP1	CTHRC1
SCUBE3	GDF15	FST	PDLIM4
TGFB1	SLC3A2	PCDH17	CLEC2B
MGP	SYT1	UACA	AKR1B1
COL1A1	NEAT1	CDC42EP3	LGALS3
VIM	GADD45A	ANK3	CTSK
PRSS3	C5orf46	UGCG	MEG3
CD59	NNMT	RGS4	MMP3
TMSB10	EFEMP1	SYNE1	SPON2
THY1	EPAS1	DSP	CFH
TUBB3	IFI16	RND3	TIMP3
SERF2	TRIB3	STAT1	S100A13
ACTB	AKR1C3	DDIT3	SPP1
IGFBP7	PCDH10	POSTN	KCTD12

Table S3. List of genes significantly up and down regulated along PC 1 and PC 2 axes of the scRNA-seq analysis. Upregulated genes become more highly expressed in the positive direction of PC 1 and PC 2, while the opposite is true for downregulated genes. These genes contribute the most to the variance seen between all measured cells.

Cluster	# of Cells	% Total
1	776	22.0%
2	648	18.4%
3	517	14.6%
4	458	13.0%
5	431	12.2%
6	219	6.2%
7	121	3.4%
8	112	3.2%
9	87	2.5%
10	75	2.1%
11	67	1.9%
12	20	0.6%
Total	3531	

Table S4. The total number of cells in each cluster of Figure 4A. Clusters were generated automatically using the Seurat software.
